# HSF1 Regulates Mevalonate and Cholesterol Biosynthesis Pathways

**DOI:** 10.3390/cancers11091363

**Published:** 2019-09-13

**Authors:** Hyeji Kang, Taerim Oh, Young Yil Bahk, Geon-Hee Kim, Sang-Yeon Kan, Dong Hoon Shin, Ji Hyung Kim, Ji-Hong Lim

**Affiliations:** 1 Department of Applied Life Sciences, Graduate School of Konkuk University, College of Biomedical & Health Science, Konkuk University, Chungju 27478, Chungbuk, Korea; kkang@kku.ac.kr (H.K.); rlarjsgml4@kku.ac.kr (G.-H.K.); hsb6477@kku.ac.kr (S.-Y.K.); 2 Diabetes and Bio-Research Center, Konkuk University, Chungju 27478, Chungbuk, Korea; 3 Department of Biomedical Chemistry, College of Biomedical & Health Science, Konkuk University, Chungju 27478, Chungbuk, Korea; dhxofla555@kku.ac.kr; 4 Department of Biotechnology, College of Biomedical & Health Science, Konkuk University, Chungju 27478, Chungbuk, Korea; byoung1@kku.ac.kr; 5 Research Institute, National Cancer Center, Department of Cancer Biomedical Science, National Cancer Center Graduate School of Cancer Science and Policy, Goyang 10408, Korea; dhshin@ncc.re.kr; 6 College of Life Sciences and Biotechnology, Korea University, Seoul 02841, Korea; jay_kim@korea.ac.kr

**Keywords:** heat shock factor 1, cholesterol, KRIBB11, simvastatin, hepatocellular carcinoma

## Abstract

Heat shock factor 1 (HSF1) is an essential transcription factor in cellular adaptation to various stresses such as heat, proteotoxic stress, metabolic stress, reactive oxygen species, and heavy metals. HSF1 promotes cancer development and progression, and increased HSF1 levels are frequently observed in multiple types of cancers. Increased activity in the mevalonate and cholesterol biosynthesis pathways, which are very important for cancer growth and progression, is observed in various cancers. However, the functional role of HSF1 in the mevalonate and cholesterol biosynthesis pathways has not yet been investigated. Here, we demonstrated that the activation of RAS-MAPK signaling through the overexpression of H-Ras^V12^ increased HSF1 expression and the cholesterol biosynthesis pathway. In addition, the activation of HSF1 was also found to increase cholesterol biosynthesis. Inversely, the suppression of HSF1 by the pharmacological inhibitor KRIBB11 and short-hairpin RNA (shRNA) reversed H-Ras^V12^-induced cholesterol biosynthesis. From the standpoint of therapeutic applications for hepatocellular carcinoma (HCC) treatment, HSF1 inhibition was shown to sensitize the antiproliferative effects of simvastatin in HCC cells. Overall, our findings demonstrate that HSF1 is a potential target for statin-based HCC treatment.

## 1. Introduction

Hepatocellular carcinoma (HCC) is the fifth most common primary liver cancer, and most cases of HCC occur in people with chronic liver disease, such as hepatitis and hepatic steatosis [1,2].

Although the main source of cholesterol is the diet, intracellular cholesterol levels are carefully regulated by mevalonate (MVA) and the cholesterol biosynthesis pathway, which is tightly controlled by sterol regulatory element-binding protein 2 (SREBP2)-mediated transcriptional programming [3]. Abnormally increased blood or intracellular cholesterol levels are associated with various human diseases, such as cardiovascular diseases, obesity, insulin resistance, hepatic steatosis, and cancer [4]. High levels of intracellular cholesterol are frequently observed in multiple types of cancer, including hepatocellular carcinoma, and are associated with cancer development, growth, invasion, metastasis, and chemoresistance [5,6,7]. Thus, statins, the inhibitors of 3-hydroxy-3-methyl-glutaryl-CoA (HMG-CoA) reductase, are widely used as cholesterol-lowering drugs for the treatment of cardiovascular diseases and cancer [8,9]. In fact, anticancer and antimetastatic effects of statins such as strovastatin, fluvastatin, and simvastatin have been observed using tissue culture and animal models in multiple types of cancer, such as hepatocellular carcinoma, breast cancer, prostate cancer, and colorectal cancer [10,11,12,13,14]. Previous animal and clinical studies have also revealed that statins decrease cancer incidence and improve cancer-related mortality [15,16,17]; however, lower therapeutic efficacy and the resistance of statins in the treatment of cardiovascular diseases and cancer via SREBP2-mediated compensation of cholesterol biosynthesis are two of the issues faced since the clinical application of statins [18,19,20].

Heat shock factor 1 (HSF1) plays a pivotal role in the proteotoxic stress response, the maintenance of proteostasis, protein translation, and cellular metabolism [21]. In response to a wide range of cellular stresses, such as heat shock, exposure to heavy metals, reactive oxygen species (ROS), and proteotoxic agents, HSF1 is activated through various post-translational modifications (PTMs), such as phosphorylation, acetylation, and sumoylation [22]. Activation of HSF1 consequently results in increased expression of heat shock response genes, such as heat shock protein family A member 6 (HSPA6), heat shock protein family A member 1B (HSPA1B), and heat shock protein family A member 1A (HSPA1A), for cellular adaptation to various cellular stresses [22]. Interestingly, previous work has identified that HSF1 regulates a wide range of targets, including metabolic enzymes as well as protein homeostasis [23]. Indeed, it was recently observed that metabolic control, including the sensing of metabolic stress and metabolic flexibility, is also regulated by HSF1. For instance, adenosine monophosphate (AMP)-activated protein kinase (AMPK), as an energetic sensor, phosphorylates and inactivates HSF1 to maintain an intracellular AMP/ATP ratio and protein quality-control machinery in response to energetic stress [24]. Similarly, HSF1 regulates hepatic mitochondrial biogenesis and mitochondrial respiration through nicotinamide phosphoribosyltransferase (NAMPT) and the nicotinamide adenine dinucleotide (NAD+) salvage pathway [25]. Interplay between HSF1 and peroxisome proliferator-activated receptor gamma coactivator 1-alpha (PGC1α) in mitochondrial metabolism (to regulate thermogenesis and the browning of white fat) has been observed [26]. In addition, decreased triglyceride (TG) and cholesterol in blood and the liver tissue in hepatic steatosis and hepatocellular carcinoma models have been observed in HSF1 knock-out mice [27]. Interestingly, recent works have shown that HSF1 is a critical factor for the growth of AKT and c-MYC-driven hepatocellular carcinoma through the maintenance of lipogenesis and cholesterol metabolism [28,29]; however, the functional role of HSF1 and its regulatory mechanisms in the mevalonate pathway and cholesterol biosynthesis have not been elucidated in-depth. Thus, we hypothesized that HSF1 may regulate the mevalonate pathway and cholesterol biosynthesis and consequently promote cell survival and growth.

Oncogenic growth signaling, such as in phosphoinositide 3-kinase (PI3K)-protein kinase B (AKT) and RAS-mitogen-activated protein kinase (MAPK), is triggered to maintain cholesterol homeostasis by activating SREBP-mediated cholesterol biosynthesis [30]. The PI3K-AKT signaling pathway, a major regulator for cell survival and proliferation in response to growth factors, can increase the mRNA and protein expression of SREBP1 and SREBP2 in response to stimulation of platelet-derived growth factor (PDGF) and vascular endothelial growth factor (VEGF) [31,32,33,34,35]. Additional reports have shown that AKT increases the stability of nuclear SREBP1a, SREBP1c, and SREBP2 by inhibiting their proteasomal degradation via F-box and tryptophan-aspartic acid (WD) repeat domain containing 7 (FBXW7), an E3 ubiquitin ligase [36]. MAP kinase ERK1/2 has also been found to phosphorylate SREBP1a at Serine 117 and SREBP2 at Serine 432 and 455 in response to insulin and PDGF; consequently, phosphorylated SREBP1a and SREBP2 are transcriptionally activated to promote fatty acid and cholesterol biosynthesis [37,38]. The hyperactivation of the oncogenic PI3K-AKT and RAS-MAPK growth signaling pathways, considered a hallmark of cancer, is driven by gain-of-function mutations, allowing for cell proliferation and survival and subsequently resulting in the development and progression of multiple types of cancer; however, the precise molecular mechanism by which RAS-MAPK activates cholesterol biosynthesis is not clearly understood.

In this study, we found that constitutively activated RAS-MAPK signaling promoted mevalonate and cholesterol biosynthesis pathways and increased the intracellular cholesterol levels by activating HSF1. Moreover, we found that improved anticancer effects in statin in HSF1 suppressed HCC cells. Overall, our results demonstrate that activated HSF1 via oncogenic RAS-MAPK signaling may be a potential target for overcoming complications arising in statin-based HCC treatment.

## 2. Results

### 2.1. An HSF1 Inhibitor, KRIBB11, Suppressed Mevalonate and Cholesterol Biosynthesis-Related Gene Expression under Lower Cholesterol Conditions in HCC Cells

**Figure 1:**
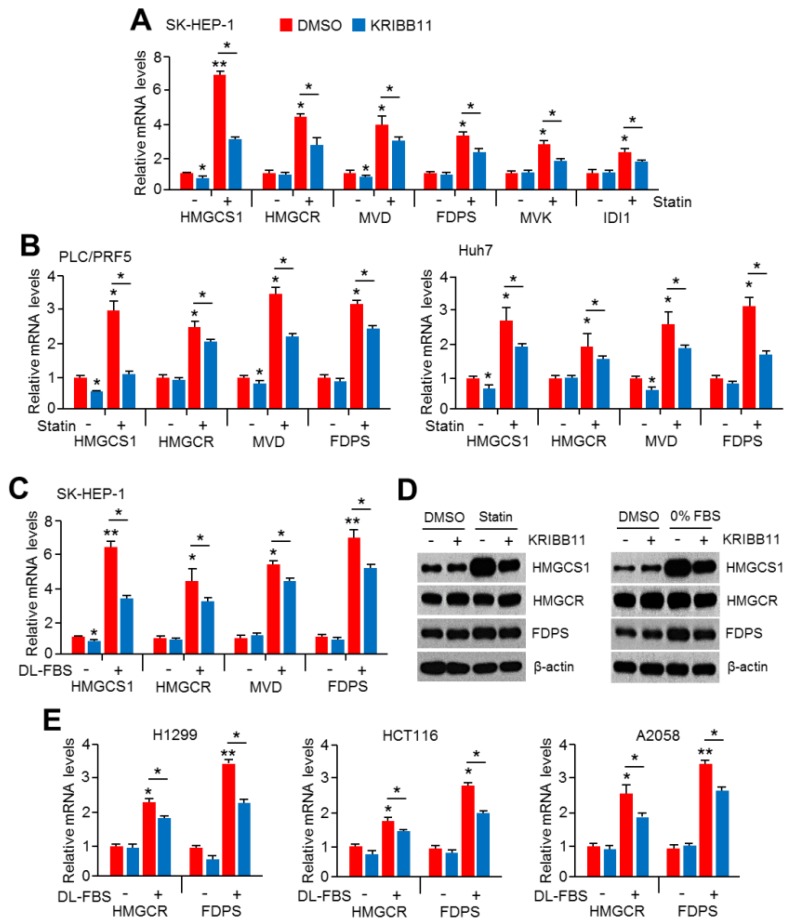
KRIBB11 attenuated mevalonate and cholesterol biosynthesis-related gene expression in hepatocellular carcinoma (HCC) cells. (**A**,**B**) SK-HEP-1, PLC/PRF5, and Huh7 cells were pretreated with KRIBB11 (2 μM) for 1 h and further treated with simvastatin (10 μM) for 24 h. The mRNA levels were measured using quantitative real-time polymerase chain reaction (qRT-PCR). The values represent the mean ± SD of two independent experiments performed in triplicate. (**C**) SK-HEP-1 cells were cultured in lipoprotein-depleted fetal bovine serum (DL-FBS) for 6 h, and then cells were further incubated with KRIBB11 (2 μM) for 24 h. The mRNA levels were measured using qRT-PCR. The values represent the mean ± SD of two independent experiments performed in triplicate. (**D**) SK-HEP-1 cells were incubated under simvastatin (10 μM) treatment or DL-FBS for 1 h or 6 h, respectively, and then KRIBB11 was further incubated for 24 h. The expression of proteins was measured by western blotting. (**E**) H1299, HCT116, and A2058 cells were cultured in lipoprotein-depleted fetal bovine serum (DL-FBS) for 6 h, and then cells were further incubated with KRIBB11 (2 μM) for 24 h. The mRNA levels were measured using qRT-PCR. The values represent the mean ± SD of two independent experiments performed in triplicate. * *p* < 0.05 and ** *p* < 0.01.

KRIBB11, a synthetic chemical and an HSF1 inhibitor, is known to have suppressive effects on heat shock response and cancer growth [39]. The functional role of HSF1 in metabolic homeostasis, such as energy expenditure and lipid metabolism, has also been demonstrated [23,26,27,28,29]. Thus, we hypothesized that HSF1 could be involved in cholesterol homeostasis. To prove this hypothesis, the possible role of HSF1 in mevalonate and cholesterol biosynthesis pathways was examined. Surprisingly, we found that increased expression of mevalonate and cholesterol biosynthesis genes caused by simvastatin treatment was significantly reversed in KRIBB11-treated SK-HEP-1 (Figure 1A), PLC/PRF5, and Huh7 (Figure 1B) cells. Furthermore, the reverse effect of KRIBB11 on mevalonate and cholesterol biosynthesis gene expression was increased by lipoprotein depletion (Figure 1C). In addition, KRIBB11 was also found to reverse simvastatin or lipoprotein depletion-induced key enzymes of cholesterol biosynthesis, in particular 3-Hydroxy-3-Methylglutaryl-CoA Synthase 1 (HMGCS1) (Figure 1D). To understand the universality of suppressive effects of KRIBB11 on cholesterol biosynthesis gene expression in other cancer types, we measured mRNA levels of *3-Hydroxy-3-Methylglutaryl-CoA Reductase* (*HMGCR*) and *farnesyl diphosphate synthase* (*FDPS*) in the absence or presence of KRIBB11 upon lipoprotein depletion in H1299 (non-small cell lung carcinoma), HCT116 (colorectal carcinoma), and A2058 (malignant melanoma) cells and found that KRIBB11 decreased *HMGCR* and *FDPS* mRNA levels induced by lipoprotein depletion in three type of cells as well as HCC cells (Figure 1E). These results indicate that HSF1 may be involved in cholesterol homeostasis in response to the blocking of cholesterol synthesis and supplementation.

### 2.2. HSF1 Knock-Down Was Sufficient to Reverse Simvastatin-Induced Gene and Enzyme Expression in Cholesterol Biosynthesis

**Figure 2:**
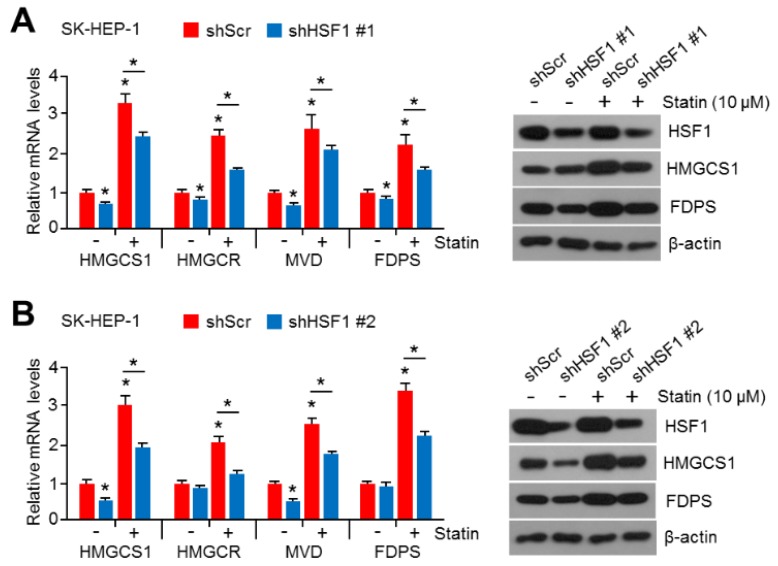
Heat shock factor 1 (HSF1) knock-down decreased mevalonate and cholesterol biosynthesis-related gene expression in HCC cells. (**A**,**B**) HSF1 knocked-down SK-HEP-1 cells were cultured under simvastatin (10 μM) treatment for 24 h. The mRNA levels were measured using qRT-PCR. The values represent the mean ± SD of two independent experiments performed in triplicate (* *p* < 0.05). The protein expression was measured by western blotting.

To rule out the abnormal expression of cholesterol biosynthesis genes caused by side effects of KRIBB11, suppressive effects of HSF1 knock-down in simvastatin-induced genes and key enzymes of cholesterol biosynthesis expression were examined. Similarly to results seen after treatment with KRIBB11, the simvastatin-induced expression of genes and key enzymes of cholesterol biosynthesis was significantly reversed in HSF1 knocked-down SK-HEP-1 cells (Figure 2A). To confirm this result, HSF1 targeting short-hairpin RNA (shRNA) that was designed from different sequences was examined (see Figure 2B). These results suggest that HSF1 is required for the expression of genes and key enzymes of cholesterol biosynthesis under decreased intracellular cholesterol levels.

### 2.3. Activation of HSF1 Increased the Expression of Cholesterol Biosynthesis-Related Genes and Enzymes

**Figure 3:**
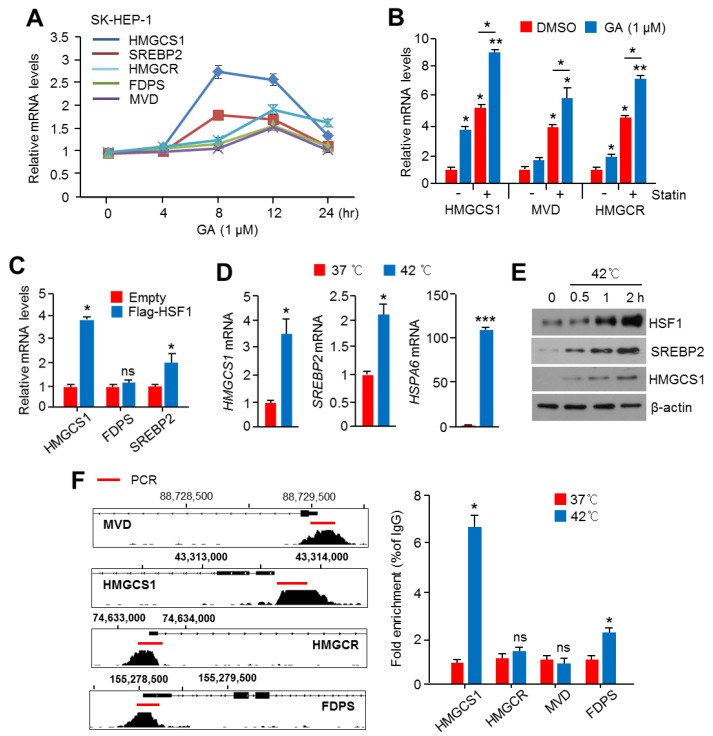
Heat Shock Factor 1 (HSF1) promoted the expression of mevalonate and cholesterol biosynthesis-related genes and proteins in HCC cells. (**A**) SK-HEP-1 cells were treated with geldanamycin (GA, 1 μM) for 4, 8, 12, and 24 h, as indicated, and mRNA levels were measured using qRT-PCR. The values represent the mean ± SD of two independent experiments performed in triplicate. (**B**) SK-HEP-1 cells were pre-incubated with simvastatin (10 μM) for 1 h, and then cells were further incubated with GA (1 μM) for 10 h. The values represent the mean ± SD of two independent experiments performed in triplicate. (**C**) Flag-tagged HSF1 was ectopically overexpressed in SK-HEP-1 cells, and then cells were incubated for 24 h. The mRNA levels were measured using qRT-PCR. The values represent the mean ± SD of two independent experiments performed in triplicate. (**D**,**E**) SK-HEP-1 cells were incubated at 37 °C or 42 °C for 1 h, and then mRNA levels were measured using qRT-PCR. The values represent the mean ± SD of two independent experiments performed in triplicate, and the protein expression was measured by western blotting. (**F**) ChIP-seq profiles of sterol regulatory element-binding protein 2 (SREBP2) enrichment indicating transcriptionally active regions of 3-Hydroxy-3-Methylglutaryl-CoA Synthase 1 (HMGCS1), 3-Hydroxy-3-Methylglutaryl-CoA Reductase (HMGCR), farnesyl diphosphate synthase (FDPS), and mevalonate diphosphate decarboxylase (MVD). Representative images obtained by ChIP-Atlas (https://chip-atlas.org/) and integrative genomic viewer (IGV, https://software.broadinstitute.org/software/igv/). SK-HEP-1 cells were incubated at 37 °C or 42 °C for 1 h and then lysed, and sonicated cell lysates were immunoprecipitated with anti-HSF1 for 16 h at 4 °C. Immunoprecipitated complexes were washed and pulled-down DNA fragments obtained. HSF1 enrichment was measured by qPCR, and fold enrichment was normalized to normal serum (IgG) control. The values represent the mean ± SD of three independent experiments performed in duplicate. * *p* < 0.05; ** *p* < 0.01; ns, not significant.

To provide direct evidence that HSF1 is an upstream factor for the regulation of cholesterol biosynthesis, we further examined the function of HSF1 in the expression of cholesterol biosynthesis-related genes and enzymes using drug treatment, genetic overexpression, and physiological stress conditions, which activate HSF1. The HSF1-activating drug, geldanamycin (GA), was found to increase cholesterol biosynthesis-related genes, in particular *HMGCS1*, *Sterol regulatory element-binding protein 2* (*SREBP2*), and *HMGCR*, in SK-HEP-1 cells (Figure 3A). In addition, GA strongly increased *HMGCS1*, *mevalonate diphosphate decarboxylase* (*MVD*), and *HMGCR* in simvastatin-treated SK-HEP-1 cells (Figure 3B). The overexpression of HSF1 also increased mRNA levels of HMGCS1 and SREBP2 under normal cholesterol conditions (Figure 3C). Because physiologically HSF1 is activated in response to heat stress, the expression of genes and key enzymes for cholesterol biosynthesis was examined in SK-HEP-1 cells under heat stress. Consistent with these results, increased *HMGCS1* and *SREBP2*, as well as the heat shock-responsive gene *HSPA6*, were observed (Figure 3D). Furthermore, increased expression of HSF1, HMGCS1, and SREBP2 in response to heat stress was observed (Figure 3E). To understand whether HSF1 could directly regulate cholesterol biosynthesis-related gene expression through promoter occupancy, we next analyzed the active transcriptional promoter regions of cholesterol biosynthesis-related genes, such as *HMGCS1*, *HMGCR*, *FDPS*, and *MVD*, which are tightly regulated by an SREBP2 transcription factor. We found a transcriptionally active promoter region with SREBP2 occupancy in these genes (shown in Figure 3F). In addition, strong occupancy of HSF1 in the promoter region of *HMGCS1* was observed in SK-HEP-1 cells exposed to heat stress (Figure 3F). Thus, these results indicate that HSF1 increased the gene expression of cholesterol biosynthesis at the transcriptionally active promoter.

### 2.4. Hyperactivation of RAS-MAPK Signaling Increased Cholesterol Biosynthesis and Intracellular Cholesterol Levels

**Figure 4:**
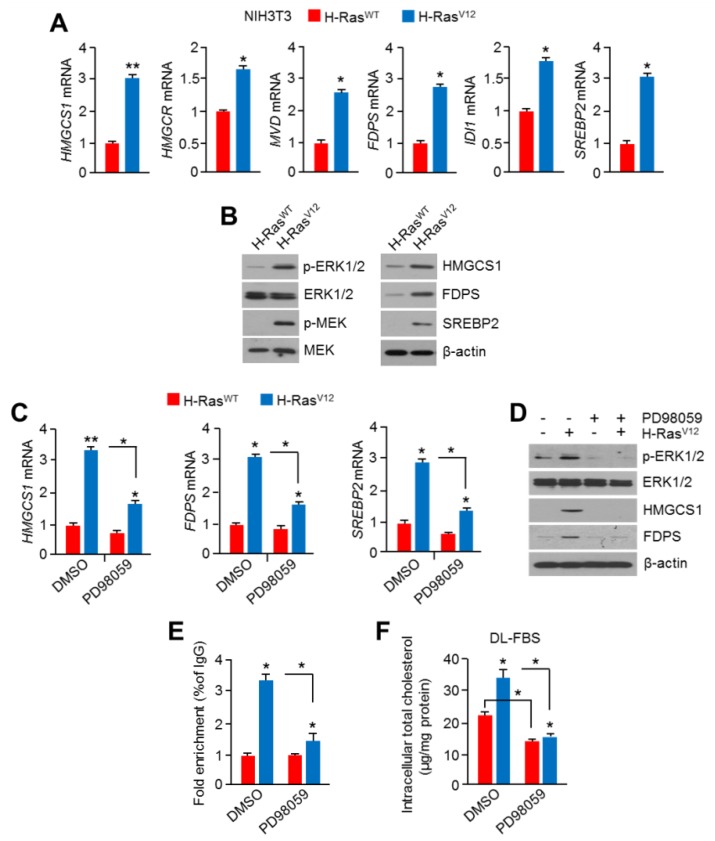
Constitutive activation of RAS-MAPK signaling promoted the mevalonate and cholesterol biosynthesis pathway. (**A**) Mevalonate and cholesterol biosynthesis-related mRNA levels were measured in H-Ras^V12^-overexpressing NIH3T3 cells. The values represent the mean ± SD of two independent experiments performed in triplicate. (**B**) Activation of MAPK signaling and cholesterol biosynthesis-related protein expression was measured in H-Ras^V12^-overexpressing NIH3T3 cells. (**C**,**D**) The MAPK inhibitor PD98059 (20 μM) was treated in H-Ras^V12^-overexpressing NIH3T3 cells for 12 h, and then mRNA and protein levels were measured. The values represent the mean ± SD of two independent experiments performed in triplicate. (**E**) H-Ras^WT^- or H-Ras^V12^-overexpressing NIH3T3 cells were cultured for 24 h in the absence or presence of PD98059 (10 μM), and then cells were used for ChIP-PCR. Cell lysates were immunoprecipitated with anti-HSF1 for 16 h at 4 °C. Immunoprecipitated complexes were washed and pulled-down DNA fragments obtained. HSF1 enrichment on the *hmgcs1* promoter was measured by qPCR, and fold enrichment was normalized to normal serum (IgG) control. The values represent the mean ± SD of three independent experiments performed in duplicate. (**F**) H-Ras^WT^- or H-Ras^V12^-overexpressing NIH3T3 cells were cultured for 24 h in a DL-FBS condition in the absence or presence of PD98059 (10 μM), and then cells were used to measure intracellular cholesterol levels. The values represent the mean ± SD of three independent experiments performed in duplicate. * *p* < 0.05 and ** *p* < 0.01.

Previously, RAS-MAPK signaling was reported to phosphorylate and activate SREBP1 and SREBP2, resulting in the promotion of fatty acid and cholesterol biosynthesis [35,36]. However, it is still unknown whether a constitutively active mutation of H-Ras into H-Ras^V12^, which is frequently observed in multiple types of cancer [40], is associated with cholesterol biosynthesis. Thus, we next examined if the overexpression of oncogenic H-Ras^V12^ causes abnormal mevalonate and cholesterol biosynthesis in normal mouse fibroblast (NIH3T3) cells. First, we found that the expression of mevalonate and cholesterol biosynthesis-related genes, such as *hmgcs1*, *hmgcr*, *mvd*, *srebp2*, and *fdps*, was significantly increased in H-Ras^V12^-expressing NIH3T3 cells (Figure 4A). In line with this, increased phosphorylation of ERK1/2 and MEK, which are directly regulated by the activation of H-Ras, and increased enzyme expression of cholesterol biosynthesis were observed in H-Ras^V12^-expressing NIH3T3 cells (Figure 4B). To confirm if cholesterol biosynthesis-related gene expression increased by H-Ras^V12^ overexpression is caused by the activation of an RAS-MAPK signaling cascade, the effects of the pharmacological inhibitor of MAPK on H-Ras^V12^-induced gene expression of cholesterol biosynthesis was tested in H-Ras^V12^-expressing NIH3T3 cells. Here, we found that PD98059, a selective MAPK inhibitor, was sufficient to suppress H-Ras^V12^-induced cholesterol biosynthesis-related gene expression (Figure 4C). Additionally, the phosphorylated ERK1/2 and cholesterol biosynthesis-related enzymes such as HMGCS1 and FDPS, which were increased by H-Ras^V12^, were dramatically diminished upon PD98059 treatment (Figure 4D). PD98059 was found to diminish HSF1 occupancy in the hmgcs1 promoter region, which was increased by H-Ras^V12^ (Figure 4E). When there is cholesterol depletion in cells, mevalonate and cholesterol biosynthesis pathways are activated to maintain intracellular cholesterol levels. Thus, we further investigated whether the activation of RAS-MAPK signaling promotes cholesterol biosynthesis under cholesterol depletion, and this phenomenon could be diminished by the MAPK inhibitor. Consistent with our hypothesis, increased intracellular cholesterol levels (by approximately 60%) were observed in H-Ras^V12^-expressing NIH3T3 cells under cholesterol depletion (Figure 4F). In addition, H-Ras^V12^-induced intracellular cholesterol levels were significantly attenuated by PD98059 treatment (Figure 4F). These results demonstrate that the activation of oncogenic H-Ras^V12^ and its downstream MAPK signaling could promote intracellular cholesterol levels through transcriptional activation of the cholesterol biosynthesis pathway.

### 2.5. HSF1 Involved Oncogenic RAS-MAPK Signaling-Induced Cholesterol Biosynthesis

**Figure 5:**
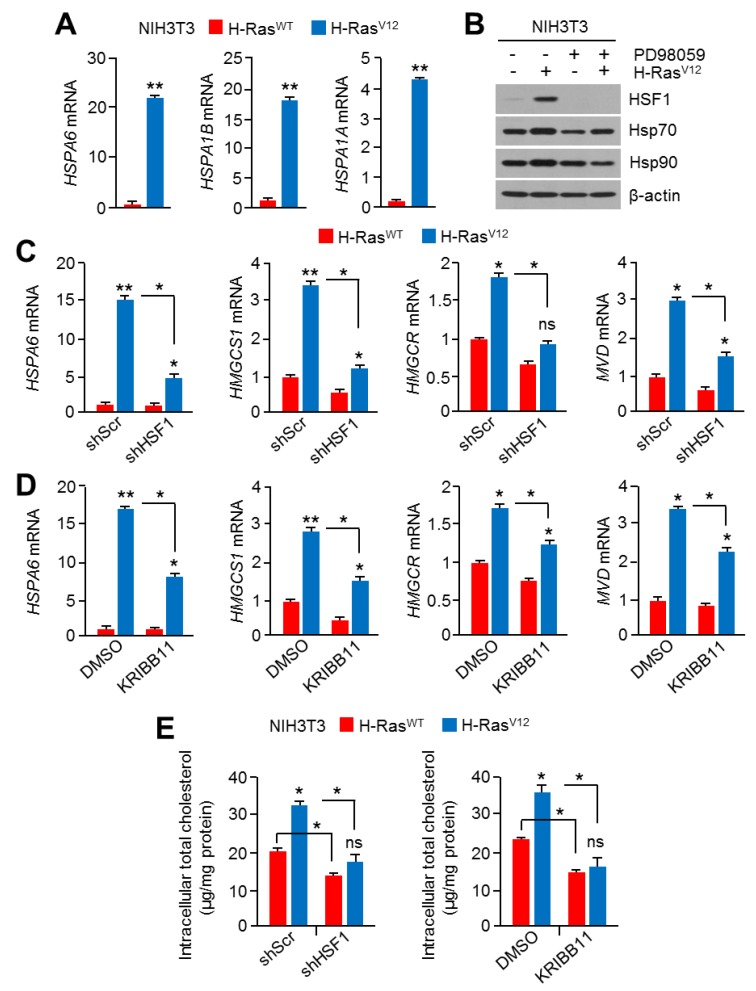
HSF1 was involved in the activation of the mevalonate and cholesterol biosynthesis pathway through RAS-MAPK signaling. (**A**) Heat shock responsive genes were measured in H-Ras^V12^-overexpressing NIH3T3 cells. The values represent the mean ± SD of two independent experiments performed in triplicate. (**B**) H-Ras^WT^- or H-Ras^V12^-overexpressing NIH3T3 cells were cultured for 24 h in the absence or presence of PD98059 (10 μM), and then protein levels were measured by western blotting. (**C**) H-Ras^WT^- or H-Ras^V12^-expressing NIH3T3 cells were infected with short-hairpin RNA (shRNA)-targeting control or HSF1-encoding lentiviral particles, and then infected cells were further incubated for 48 h. The mRNA levels were measured using qRT-PCR. The values represent the mean ± SD of three independent experiments performed in duplicate. (**D**) H-Ras^WT^- or H-Ras^V12^-expressing NIH3T3 cells were incubated for 24 h in the absence or presence of KRIBB11 (5 μM), and then mRNA levels were measured. The values represent the mean ± SD of three independent experiments performed in duplicate. (**E**) H-Ras^WT^- or H-Ras^V12^-overexpressing NIH3T3 cells were cultured for 24 h in a DL-FBS condition in the absence or presence of HSF1 knocking-down shRNA or KRIBB11 (5 μM), and then cells were used to measure intracellular cholesterol levels. The values represent the mean ± SD of three independent experiments performed in duplicate. * *p* < 0.05; ** *p* < 0.01; ns, not significant.

Although RAS-MAPK signaling increases HSF1 phosphorylation and the activation of cancer development and progression through adaptation to cellular proteotoxic and metabolic stress [21,22,41], the correlation between HSF1 and cholesterol biosynthesis induced by oncogenic RAS-MAPK signaling during cancer development and progression has not been investigated. Thus, we examined whether HSF1 participates in RAS-MAPK signaling-induced cholesterol biosynthesis. Increased expression of heat shock-responsive HSF1 targeting downstream genes, such as hspa6 and hspa1b, was confirmed in H-Ras^V12^-expressing NIH3T3 cells (Figure 5A). In addition, H-Ras^V12^ increased heat shock proteins, such as Hsp70 and Hsp90, and PD98059 diminished H-Ras^V12^-induced heat shock proteins (Figure 5B), indicating that RAS-MAPK signaling activated HSF1 and its heat shock-responsive downstream targets. To examine if HSF1 is a component of linear axis to H-Ras^V12^-induced cholesterol biosynthesis, reverse effects of HSF1 knock-down in H-Ras^V12^-induced cholesterol biosynthesis-related gene expression was tested. Interestingly, cholesterol biosynthesis-related gene expression induced by H-Ras^V12^ was significantly attenuated in HSF1 knocked-down cells (Figure 5C). KRIBB11 was also found to reduce H-Ras^V12^-induced cholesterol biosynthesis-related gene expression (Figure 5D). Consistent with these findings, increased intracellular cholesterol levels by H-Ras^V12^ were also diminished by HSF1 knock-down and KRIBB11 treatment (Figure 5E). Overall, these results reveal that HSF1 acted as an essential factor in the activation of cholesterol biosynthesis through RAS-MAPK signaling.

### 2.6. Suppression of HSF1 Sensitively Attenuated Cell Growth under Cholesterol Depletion in HCC Cells

**Figure 6:**
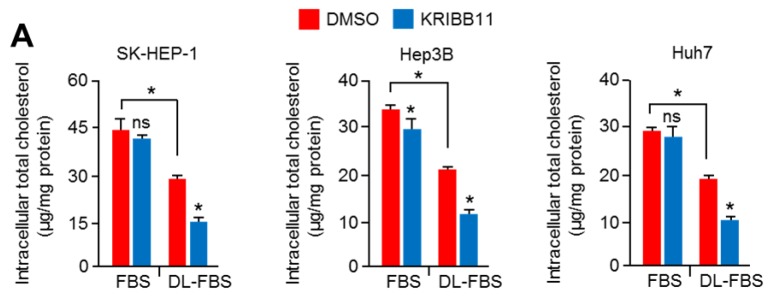

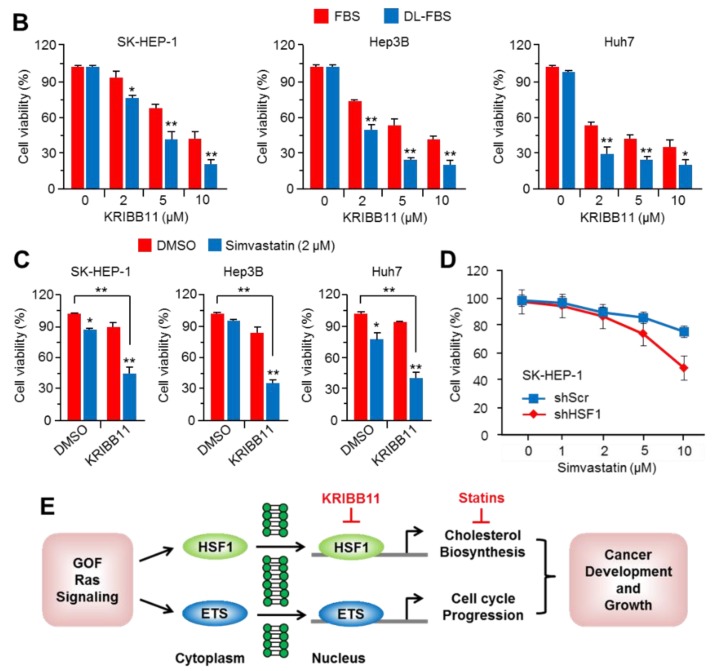
HSF1 inhibition sensitized the suppression of HCC cell growth in cholesterol-depleted conditions. (**A**) SK-HEP-1, Hep3B, and Huh7 were incubated for 24 h in normal FBS or DL-FBS in the absence or presence of KRIBB11 (5 μM), and then cells were used to measure intracellular cholesterol levels. The values represent the mean ± SD of three independent experiments performed in duplicate. (**B**) SK-HEP-1, Hep3B, and Huh7 were incubated for 48 h in normal FBS or DL-FBS in the absence or presence of various concentrations of KRIBB11 (2, 5, and 10 μM), as indicated. Cell viability was measured by crystal violet staining. The values represent the mean ± SD from three independent experiments performed in duplicate. (**C**) SK-HEP-1, Hep3B, and Huh7 were incubated for 48 h in Dimethyl sulfoxide (DMSO) (as a control) or simvastatin (2 μM) in the absence or presence of KRIBB11 (5 μM). The values represent the mean ± SD from three independent experiments performed in duplicate. (**D**) HSF1 knocked-down SK-HEP-1 cells were incubated with various concentrations of simvastatin (1, 2, 5, and 10 μM) for 48 h, as indicated. The values represent the mean ± SD from three independent experiments performed in duplicate. (**E**) Proposed molecular mechanism by which HSF1 and erythroblast transformation-specific (ETS) transcription factor activated by gain-of-function (GOF) RAS-MAPK signaling promotes cholesterol biosynthesis and cell cycle progression, and this regulatory axis may be involved in cancer development and progression. * *p* < 0.05; ** *p* < 0.01; ns, not significant.

It is becoming clear that abnormal mevalonate and cholesterol biosynthesis pathways are potential therapeutic targets for cancer treatment, since they are often observed in multiple types of cancers and are involved in cancer growth and progression [5,7,42]. Given that HSF1 promotes cholesterol biosynthesis under cholesterol depletion, we examined the functional role of HSF1 in cholesterol biosynthesis and cell growth in HCC cells under cholesterol depletion or normal cholesterol supplementation. Initially, KRIBB11 was found to decrease intracellular cholesterol levels in SK-HEP-1, Hep3B, and Huh7 cells cultured in cholesterol depletion (Figure 6A), indicating that the inhibition of HSF1 caused the disruption of cholesterol homeostasis. In addition, stronger antiproliferative effects of KRIBB11 were observed in SK-HEP-1, Hep3B, and Huh7 cells in cholesterol depletion compared to normal cholesterol supplementation (Figure 6B). Thus, these results indicate that KRIBB11-treated HCC cells may collapse in cellular division due to decreased intracellular cholesterol, which is an essential building block for cellular proliferation. A clinical limitation of statin-based cardiovascular and cancer therapy is that mevalonate and cholesterol biosynthesis pathways are reactivated through multivalent feedback mechanisms [18]; hence, we examined the synergized antiproliferative effects of the combination of simvastatin and KRIBB11 to provide a plausible therapeutic strategy. As we speculated, KRIBB11 was sufficient to sensitize antiproliferative effects of simvastatin in SK-HEP-1, Hep3B, and Huh7 cells (Figure 6C). Consistently, sensitized antiproliferative effects of simvastatin were also observed in HSF1 knocked-down SK-HEP-1 cells (Figure 6D). Thus, taken together, our findings demonstrate that HSF1 may be a potential target for statin-based treatment and cancer management through a low-cholesterol diet in HCC patients.

## 3. Discussion

It is becoming clear that heat shock factor 1 (HSF1) is a pivotal transcription factor for adaptation to various cellular stresses, such as proteotoxic and metabolic stresses [22]. Although accumulated evidence has provided the possibility that HSF1 may be associated with cholesterol metabolism and its related human diseases, including cancer, the correlation between HSF1 and cholesterol metabolism in cancer development and progression has not yet been identified. Recently, Jin et al. demonstrated that triglyceride (TG) and cholesterol were lower in blood and liver tissue, and a preventive effect on hepatic steatosis was observed in HSF1 knock-out mice [27]. Furthermore, reduced mRNA levels associated with lipogenesis (*fas*, *acc,* and *scd1*) and lipogenic factors (*srebp1c*, *pparγ1*, *pparγ2*, and *c/ebpβ*) were observed in diethylnitrosamine (DEN)-induced hepatic steatosis and hepatocellular carcinoma (HCC) model-driven HSF1 knock-out mice [27]. In addition, the suppression of lipogenesis and cholesterol metabolism by HSF1 suppression in an AKT and c-MYC-driven hepatocellular carcinoma model has been observed [28,29]. These backgrounds indicate that HSF1 could regulate the mevalonate pathway and cholesterol biosynthesis to maintain the homeostasis of mevalonate metabolites and cholesterol in the liver. Consistently, we observed that the activation of HSF1 by heat shock stress, the actions of a pharmacological activator (geldanamycin), and the ectopic overexpression of HSF1 significantly promoted mevalonate pathway- and cholesterol biosynthesis-related genes, in particular *HMGCS1* expression in hepatocellular carcinoma cells. Thus, our findings indicate that abnormally increased mevalonate pathways and cholesterol biosynthesis through the transcriptional activation of HSF1 might be partly involved in the development of HCC and its progression. Contrary to our findings, heat shock protein 90 (HSP90) was found to stabilize and activate sterol-responsive element binding proteins (SREBPs) through direct interaction between SREBPs and the SREBP cleavage-activating protein (SCAP). Furthermore, pharmacological inhibition of HSP90 by 17-AAG increased genes related to fatty acid and cholesterol biosynthesis, such as 3-hydroxy-3-methylglutaryl-CoA synthase 1 (*HMGCS1*), fatty acid synthase (*FASN*), and 3-hydroxy-3-methylglutaryl-CoA reductase (*HMGCR*), in HepG2 cells [43]. These findings thus need further elucidation.

The hyperactivation of the RAS-MAPK signaling pathway promotes cellular processes, including cell cycle progression, proliferation, differentiation, and cell survival [21]. Gain-of-function mutations in genes of the RAS-MAPK pathway are observed in approximately 30% of all human cancers [40,44]. A recent study using the Cancer Genome Atlas (TCGA) database found that several oncogenic signaling pathways, such as the PI3K-AKT and RAS-MAPK pathways, increase cholesterol biosynthesis-related gene expression in several types of cancer [7]. Consistently, we also observed that a constitutively activated RAS-MAPK pathway through the overexpression of H-Ras^V12^ increased mevalonate pathway- and cholesterol biosynthesis-related genes and enzyme expression in NIH3T3 cells. In addition, increased intracellular cholesterol levels were observed in H-Ras^V12^-expressing NIH3T3 cells, and H-Ras^V12^-induced intracellular cholesterol levels were reversed by PD98059, a MAPK inhibitor. Thus, a possible therapeutic strategy through the concomitant suppression of the PI3K-AKT and RAS-MAPK signaling pathways to target abnormally increased cholesterol metabolism-associated cancers could be suggested.

MEK, a component kinase of the RAS-MAPK cascade, as an inhibitor has been found to inhibit HSF1 Ser326 phosphorylation and its transcriptional activity, consequently suppress heat shock responses and cellular metabolism [21]. Although HSF1 is not considered to be a canonical oncogene, several reports have shown that HSF1 knock-out mice are more resistant to tumor development induced by carcinogenic, mutant p53, and oncogenic H-Ras [45]. Abnormally increased HSF1 is frequently observed in multiple types of cancer, including HCC, and is associated with invasion and metastasis, and thus it is considered to be a prognostic marker of HCC [46]. However, the precise molecular mechanisms by which increased and activated HSF1 via RAS-MAPK signaling causes tumor development and progression is still poorly understood. Here, we observed that the hyperactivation of RAS-MAPK signaling through the overexpression of H-Ras^V12^ increased HSF1 and cholesterol biosynthesis-related gene expression and intracellular cholesterol levels, as well as heat shock-responsive genes. Moreover, the suppression of HSF1 was found to rescue cholesterol biosynthesis-related gene expression and intracellular cholesterol levels. In aggregate, these results indicate that HSF1 might be required for the activation of the mevalonate pathway and cholesterol biosynthesis through oncogenic RAS-MAPK signaling.

It is still unclear how HSF1 mechanistically regulates intracellular cholesterol homeostasis. Interestingly, previous work has shown that a gain-of-function mutant of p53 enhances the mRNA expression of genes associated with the mevalonate pathway and cholesterol biosynthesis, such as *HMGCR*, *mevalonate kinase* (*MVK*), *farnesyl diphosphate synthase* (*FDPS*), and *squalene epoxidase* (*SQLE*), by cooperating with SREBP2, resulting in the promotion of cancer progression [47,48]. In fact, increasing evidences have shown that the mutant p53 provides advantages of cellular growth and survival of HCC cells [49,50]. Recently, mutant p53 was also found to interact with HSF1 and subsequently govern the adaptation of cancer cells to proteotoxic and metabolic stress [51]. Based on these backgrounds, a plausible model could be suggested that shows that the interplay between HSF1 and mutant p53 is partly involved in the mevalonate pathway and cholesterol biosynthesis to promote cancer development and progression.

Abnormal lipogenesis and cholesterol metabolism are frequently observed in multiple types of cancer, including HCC, and are associated with cancer development and progression [4,5,6,7]. In fact, recent work has shown that the concomitant inhibition of fatty acid synthase (FASN)-mediated lipogenesis and HMGCR-mediated cholesterol biosynthesis successfully suppresses the growth of human HCC [52], indicating that abnormal lipogenesis and cholesterol metabolism may be potential targets for HCC treatment. Consistent with this, it is becoming clear that statins, which are cholesterol-lowering drugs, exhibit anticancer effects through cell cycle arrest, apoptotic cell death, and cell stemness suppression in several types of cancer [10,11,12,13,14]. In fact, emerging clinical evidence has shown the beneficial effects of statins to reduce cancer-related mortality in multiple cancer types, such as HCC, breast cancer, lung cancer, prostate cancer, colorectal cancer, and kidney cancer [15,16,53,54,55,56,57,58]. However, a paradoxical feedback mechanism by which statins induce HMGCR stability and SREBP2-mediated compensation of cholesterol biosynthesis often causes lower therapeutic efficacy of statins in the treatment of heart disease and cancer [18,19,20]. Increased farnesyl diphosphate (FPP) and geranylgeranyl diphosphate (GGPP) through the activation of the mevalonate pathway induces protein prenylation, a lipid post-translational modification, which is required for cellular transformation, cancer stemness, and the growth and suppression of apoptosis by activating oncogenic signaling proteins, including RAS superfamily members [59]. Indeed, several reports have shown that the diminished anticancer efficacy of statin through the additional supplementation of FPP and GGPP was observed in osteosarcoma, glioblastoma, and leukemia [60,61,62]. The production of FPP and GGPP through the mevalonate pathway is activated in multiple types of cancer, and results in resistance to the anticancer efficacy of statins [5,6,7]: a more potent inhibition of the mevalonate and cholesterol synthetic pathways may improve the anticancer efficacy of statins. In the present study, we found that KRIBB11 (an HSF1 inhibitor) and HSF1 knock-down suppressed mevalonate pathway- and cholesterol biosynthesis-related genes and enzyme expression upon simvastatin treatment in HCC cells. Moreover, KRIBB11 and HSF1 knock-down significantly enhanced the antiproliferative effects of simvastatin by approximately 50% in HCC cells. These results indicate that the suppression of HSF1 is a potential therapeutic strategy to improve statin-based HCC treatment.

Thus, we found the following: (1) the oncogenic gain-of-function mutation in the RAS-MAPK signaling pathway increased HSF1; (2) increased HSF1 through RAS-MAPK signaling involved intracellular cholesterol homeostasis by activating the cholesterol biosynthesis pathway; and (3) KRIBB11, an HSF1 inhibitor, suppressed cholesterol biosynthesis and sensitized the antiproliferative effects of statin in HCC cells (Figure 6E). Taken together, our results suggest that HSF1 may be a potential target for HCC treatment.

## 4. Materials and Methods

### 4.1. Reagents and Antibodies

Geldanamycin (sc-200617), KRIBB11 (385570), and simvastatin (S6196) were purchased from Santa Cruz Biotechnology (Dallas, TX, USA), Millipore (Burlington, MA, USA), and Sigma Aldrich (St. Louis, MO, USA), respectively. The antibodies for HSF1 (CST-12972), HMGCS1 (CST-36877), MEK (CST-9122), phospho-MEK (CST-9154), and phospho-ERK1/2 (CST-4370) were purchased from Cell Signaling Technology (Danvers, MA, USA). HMGCR (ab174830), FDPS (ab189874), and SREBP2 (ab30682) antibodies were bought from Abcam (Cambridge, MA, USA), and β-actin (sc-47778) and ERK1/2 (sc-94) were purchased from Santa Cruz Biotechnology (Dallas, TX, USA).

### 4.2. Western Blotting

Cells were lysed with NP-40 buffer, as previously described [10]. Protein samples were separated according to molecular weight by SDS-PAGE, and the separated proteins were transferred onto polyvinylidene difluoride (PVDF) membranes (Millipore, Burlington, MA, USA). The transferred membranes were probed overnight at 4 °C with primary antibodies (1:1000–1:5000). After primary antibody reaction, horseradish peroxidase (HRP)-conjugated secondary antibodies (1:10,000) were added for 1 h at room temperature. Protein expression was visualized and analyzed using an Enhanced Chemiluminescence (ECL) Prime kit (GE Healthcare, Pittsburgh, PA, USA). Detailed information can be found at Supplementary Figure S1 and Table S1.

### 4.3. Cell Culture, Plasmids, and Generation of Stable Cell Lines

SK-HEP-1, PLC/PRF5, Hep3B, Huh7, H1299, HCT116, and A2058 cells were obtained from the Korean Cell Line Bank (Seoul, Korea) and cultured in Dulbecco’s Modified Eagle’s Medium (DMEM), Roswell Park Memorial Institute (RPMI) 1640, and Minimum Essential Medium Eagle alpha medium (MEM-α) containing 10% fetal bovine serum (FBS) and antibiotics. For cholesterol depletion, lipoprotein-depleted FBS (DL-FBS, Kalen Biomedical, Germantown, MD, USA) was used, and pLKO.1-shRNA-HSF1 (TRCN0000007482 and TRCN0000007480, Sigma Aldrich) was used to generate HSF1 knock-down SK-HEP-1 cells. In addition, pLKO.1-shRNA-HSF1 (TRCN0000008502 and TRCN0000008504, Sigma Aldrich) was used to generate HSF1 knock-down NIH3T3 cells. To produce lentiviral particles containing shRNA against HSF1, pLKO.1-shRNA vector, envelope vector (pMD2.G), and packaging vector (psPAX2) were transfected into the HEK293T cells using Polyfect reagent (Qiagen, Venlo, Netherlands), and then these lentiviral particles were infected into SK-HEP-1 and NIH3T3 cells, as previously described [63]. For the generation of H-Ras^V12^-overexpressing NIH3T3 cells, transfections were performed using FuGENE 6 Reagent (Roche Molecular Biochemicals, Nutley, USA) according to the manufacturer’s instructions. After the first G418 sulfate selection (800 mg/mL), individual G418 sulfate-resistant clones were selected, amplified, and screened by measuring phospho-ERK1/2 and phospho-MEK expressions for the stable expression of H-Ras^V12^, as previously described [64]. Flag-tagged HSF1 (Addgene plasmid # 32537) was a gift from Stuart Calderwood [65].

### 4.4. Measurement of mRNA Expression

Total RNA was extracted by using TRIzol (Invitrogen, Carlsbad, CA, USA) and reverse-transcribed into cDNA using a high-capacity cDNA reverse transcription kit (Applied Biosystems, Waltham, MA, USA). Quantitative real-time PCR was performed by using SYBR Green PCR Master Mix (Applied Biosystems, Waltham, MA, USA) according to the manufacturer’s protocol. The primer sequences used in the experiment are shown in Table 1.

### 4.5. Chromatin Immunoprecipitation (ChIP) Assay

SK-HEP-1 cells were exposed to heat stress at 42 °C for 1 h prior to chromatin immunoprecipitation (ChIP). A ChIP assay was performed using a ChIP assay kit (Millipore, Burlington, MA, USA) according to the manufacturer’s instructions and slight modifications. Cells were incubated with 4% formaldehyde for 10 min at room temperature to cross-link the proteins to the DNA. Glycine solution was added into the cross-linked cells and incubated for 5 min to quench formaldehyde. To shear DNA, harvested cells were sonicated until a DNA fragment size of 200–500 bp was reached. The sonicated cell lysates were precleared using protein A/G-agarose beads (Santa Cruz Biotechnology) for 1 h. Equal amounts of precleared cell lysates were incubated with normal serum (IgG) or anti-HSF1 (CST-12972, Cell Signaling Technology, Danvers, MA, USA) overnight at 4 °C, and then immunocomplexes were washed and RNA and proteins were removed using RNase A and proteinase K (Millipore, Burlington, MA, USA). Quantitative real-time PCR was performed by using SYBR Green Mixture (Applied Biosystems, Waltham, MA, USA) to analyze the promoter occupancy, as previously described [66]. The primer sequences used in the experiment are shown in Table 2.

### 4.6. Measurement of Intracellular Total Cholesterol

Intracellular cholesterol levels in SK-HEP-1, Hep3B, and Huh7 cells were measured using the Amplex Red cholesterol assay kit (Invitrogen, Waltham, MA, USA) in accordance with the manufacturer’s protocols, as previously described [10]. Cells were harvested and frozen at −70 °C overnight. The frozen cells were then incubated with reaction buffer for 30 min on ice and then sonicated to disrupt the cellular membrane. Samples (50 μL) were mixed with an equal volume of Amplex Red reagent containing HRP, cholesterol oxidase, and cholesterol esterase, and then the mixture was left for 30 min at 37 °C. The fluorescence intensity was measured using a fluorescence microplate reader (BioTek, Winooski, VT, USA) at ex/em wavelengths of 530/590. The cholesterol levels were normalized using protein concentrations determined by a Bradford assay.

### 4.7. Cell Viability

Cell viability was measured by crystal violet staining, as previously described [10]. SK-HEP-1, Hep3B, and Huh7 cells were seeded into 24-well tissue culture dishes with normal lipoprotein-containing FBS or lipoprotein-depleted FBS in the absence or presence of KRIBB11, HSF1 knock-down, or simvastatin. Cultured cells were washed and fixed with phosphate-buffered saline (PBS) and 4% paraformaldehyde, and then cells were stained using crystal violet solution for 20 min at room temperature. The optical density of crystal violet-stained cells was measured at 570 nm by using an absorbance reader (BioTek, Winooski, VT, USA) (OD570).

### 4.8. Statistical Analysis

Data are represented as the mean ± standard deviation (SD). All statistical analyses were performed using a two-tailed Student’s *t*-test and one-way ANOVA with Tukey’s post hoc test. A *p*-value < 0.05 was considered statistically significant.

## 5. Conclusions

In the present study, we found that activation of HSF1 via oncogenic H-Ras^V12^ signaling promoted cholesterol biosynthesis and that the suppression of HSF1 sensitized the anticancer effects of simvastatin in HCC cells. Collectively, our work demonstrates that targeting HSF1 can be a promising strategy that can be used synergistically with statins for the treatment of patients with HCC.

## Figures and Tables

**Table 1:** Primer sequences for q-PCR.

Gene	Forward Primer	Reverse Primer
*HMGCS1* (h)	TGGCAGGGAGTCTTGGTACT	TCCCACTCCAAATGATGACA
*HMGCR* (h)	GATGGGAGGCCACAAAGAG	TTCGGTGGCCTCTAGTGAGA
*MVD* (h)	AACATCGCGGTCATCAAGTA	TTAACTGGTCCTGGTGCAGA
*FDPS* (h)	AGCCAAGGAAACAGGATG	TCCATGATGTCATCTGCCAC
*MVK* (h)	GAGGTCGCCAGCTCTCCA	GAACTTGAGCAGCCTGTTTCTGA
*HMGCS1* (m)	TGGCAGGGAGTCTTGGTACT	TCCCACTCCAAATGATGACA
*HMGCR* (m)	TGTCCCCACTATGACTTCCC	TCGGTGGCCTCTAGTGAGAT
*MVD* (m)	ATGGCCTCAGAAAAGCCTCAG	TGGTCGTTTTTAGCTGGTCCT
*FDPS* (m)	GGAGGTCCTAGAGTACAATGCC	AAGCCTGGAGCAGTTCTACAC
*IDI1* (m)	GGTTCAGCTTCTAGCGGAGA	TCGCCTGGGTTACTTAATGG
*HSPA6* (m)	CCAAATGCAAGACAAGTGTCG	TTCTAGCTTTGGAGGGAAAG
*HSPA1B* (m)	CCCTACCATTGAGGAGGTG	AAACTCGTACAGAAGGTGGC
*HSPA1A* (m)	GGCAAGATCAGCGAGGCC	TCTCTGCATGTAGAAACCGC

**Table 2:** Primer sequences for chromatin immunoprecipitation (ChIP)-PCR.

Gene	Forward Primer	Reverse Primer
***HMGCS1***	CGCTGGAGAGATGGTCAAAT	CTTTCAGGTGAAGCCTAGTTCT
***HMGCR***	GAGAACATAAGCAGGGAGGTAAA	TTCCTGTGCGAACCTTACAG
***MVD***	AAGGTGGGCGTCTCAAATAC	CCTGTACGTTTCACAGGAGAAG
***FDPS***	AGCTGCCCAGGAAGATAATG	CAATGGAAGGGCTGAAGTCTA

## Supplementary Materials

The following are available online at https://www.mdpi.com/2072-6694/11/9/1363/s1, Figure S1: Whole Scan of Western Blots Film, Table S1: Densitometry analysis of Western Blots. All band intensities were normalized to control band in each experimental group.
