# Supplementary material for: HSF1 Regulates Mevalonate and Cholesterol Biosynthesis Pathways
Source: Cancers (Basel). 2019 Sep 13;11(9):1363. doi: 10.3390/cancers11091363 (PMC6769575; doi:10.3390/cancers11091363)
Supplement: Supplementary file 1 [file cancers-11-01363-s001.pdf]

Supplementary Materials: HSF1 Regulates Mevalonate and Cholesterol Biosynthesis Pathways

Hyeji Kang, Taerim Oh, Young Yil Bahk, Geon-Hee Kim, Sang-Yeon Kan, Dong Hoon Shin, Ji Hyung Kim and Ji-Hong Lim

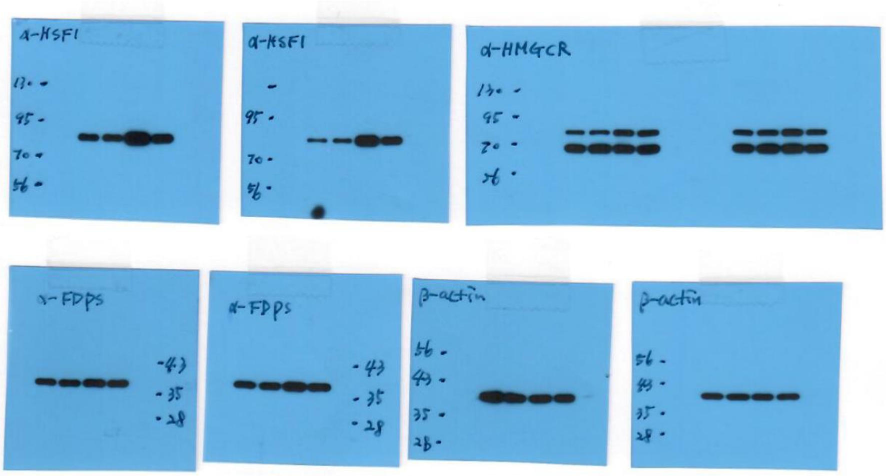

Figure 1D

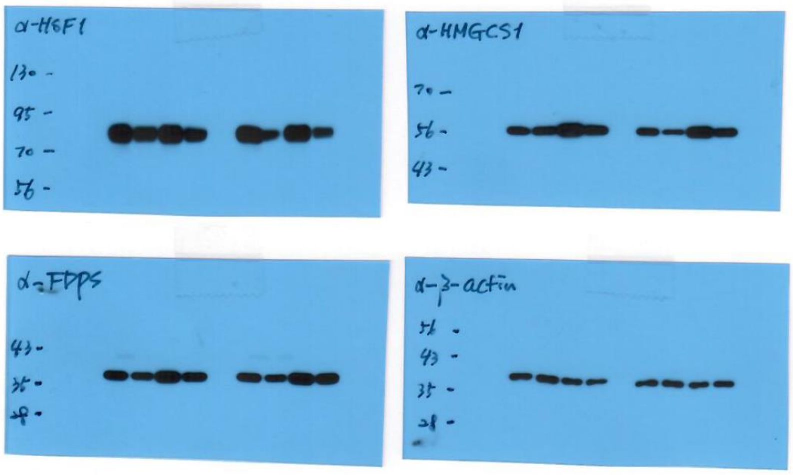

Figure 2A,B

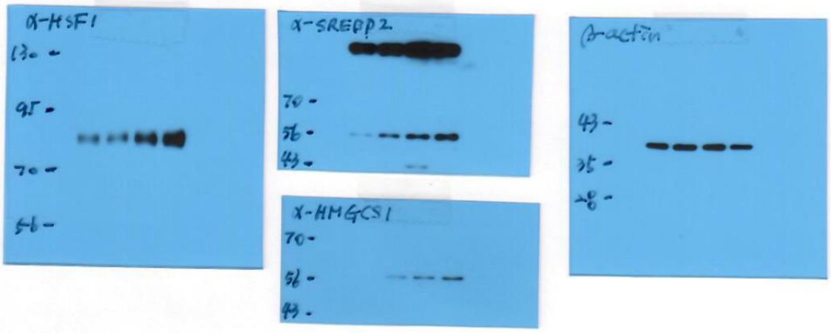

Figure 3E

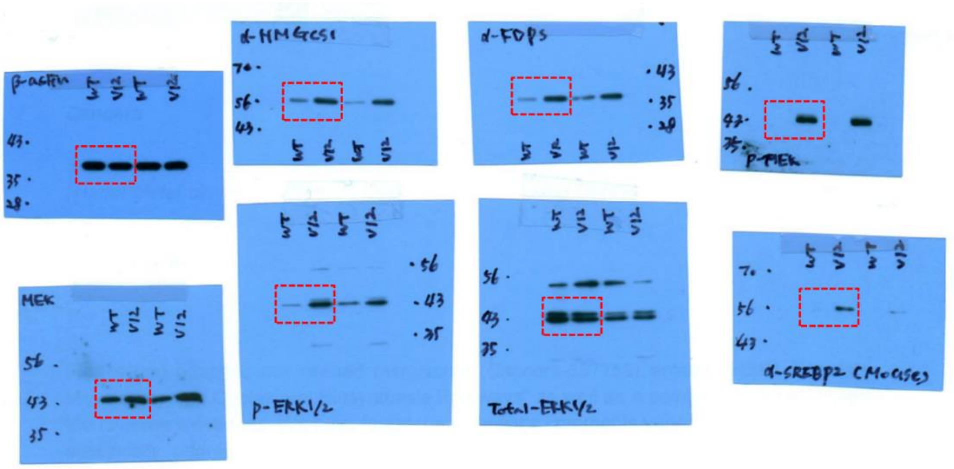

Figure 4B

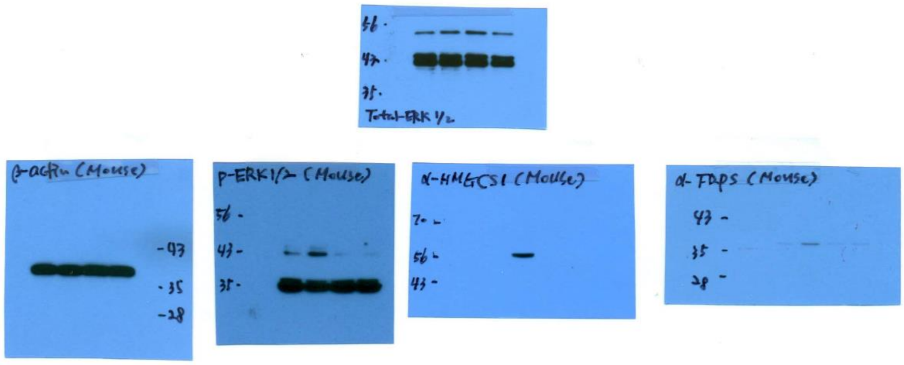

Figure 4D

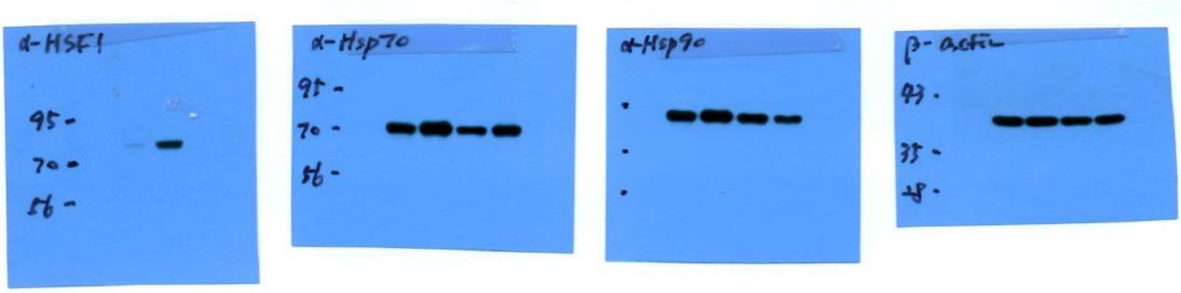

Figure 5B

Figure S1. Whole Scan of Western Blots Film.

Table S1. Densitometry analysis of Western Blots. All band intensities were normalized to control band in each experimental group.

	**Figure 1D**							
	DMSO		Statin		DMSO		Statin	
KRIBB11	-	+	-	+	-	+	-	+
HMGCS1	1	0.95	8.4	3.6	1	1.2	9.8	4.1
HMGCR	1	1.6	1.1	1.3	1	1.5	1.4	1.1
FDPS	1	1.3	1.7	1.5	1	1.2	2.1	1.5
β-actin	1	0.9	0.9	0.8	1	1.1	1	1
	Figure 2A				Figure 2B			
	**DMSO**		**Simvastatin**		**DMSO**		**Simvastatin**	
shRNA	Scr	HSF1#1	Scr	HSF1#1	Scr	HSF1#2	Scr	HSF1#2
HSF1	1	0.7	1	0.6	1	0.3	1	0.4
HMGCS1	1	1.1	2.4	1.5	1	0.4	2.8	1.7
FDPS	1	0.8	1.9	1.1	1	0.8	1.7	1.5
β-actin	1	1.1	0.9	0.8	1	1	0.9	0.9
	**Figure 3E**							
Heat Shock (hour)	0		0.5 h		1 h		2 h	
HSF1	1		1.1		2.2		4.9	
SREBP2	1		1.9		2.3		3.1	
HMGCS1	Not detected		1		1.8		2.3	
β-actin	1		1.1		1		0.8	
	**Figure 4B**							
H-Ras	H-Ras^{WT}				H-Ras^{V12}			
p-ERK1/2	1				3.2			
ERK1/2	1				1.1			
p-MEK	Not detected				1			
MEK	1				1.4			
HMGCS1	1				2.9			
FDPS	1				3.1			
SREBP2	Not detected				1			
β-actin	1				1			
	**Figure 4D**							
H-Ras	H-Ras^{WT}		H-Ras^{V12}		H-Ras^{WT}		H-Ras^{V12}	
PD98059	-		-		+		+	
p-ERK1/2	1		1.9		0.2		0.2	
ERK1/2	1		1		1		0.8	
HMGCS1	Not detected		1		Not detected		Not detected	
FDPS	1		2.8		0.8		1.2	
β-actin	1		1		1		1	
	**Figure 5B**							
H-Ras	H-Ras^{WT}		H-Ras^{V12}		H-Ras^{WT}		H-Ras^{V12}	
PD98059	-		-		+		+	
HSF1	Not detected		1		Not detected		Not detected	
Hsp70	1		1.6		0.8		0.9	
Hsp90	1		1.8		1		0.8	
β-actin	1		1		0.9		0.9	

© 2019 by the authors. Licensee MDPI, Basel, Switzerland. This article is an open access article distributed under the terms and conditions of the Creative Commons Attribution (CC BY) license (http://creativecommons.org/licenses/by/4.0/).
